# EPIDEMIOLOGICAL PROFILE OF PATIENTS WITH ANKLE FRACTURE TREATED IN A TERTIARY LEVEL HOSPITAL

**DOI:** 10.1590/1413-785220243206e282171

**Published:** 2025-01-10

**Authors:** Adriana Cano Buric da Silva, José Carlos Baldocchi Pontin, Ana Paula Cortes Damasceno, Lucas Simões Arrebola, Cairo dos Reis Souza, Marcos Vinicius Malheiros Luzo

**Affiliations:** 1. Universidade Federal de São Paulo, Escola Paulista de Medicina, São Paulo, SP, Brazil.; 2. Universidade Federal de São Paulo, Instituto Saúde e Sociedade da Baixada Santista, Departamento de Ciências do Movimento Humano e Reabilitação, Santos, SP, Brazil.; 3. Universidade Federal de São Paulo, Escola Paulista de Medicina, Departamento de Ortopedia e Traumatologia, São Paulo, SP, Brazil.

**Keywords:** Bone Fractures, Ankle, Orthopedic Procedures, Fraturas ósseas, Tornozelo, Procedimentos Ortopédicos

## Abstract

Objective: To evaluate the epidemiological profile of patients diagnosed with ankle fracture admitted to a tertiary hospital. Methods: Retrospective Cross-Sectional Observational Study. Inclusion Criteria: Individuals aged ≥18 (eighteen) years, diagnosed with ankle fracture, who underwent surgical and/or conservative treatment at a tertiary hospital in the city of São Paulo. Exclusion Criteria: Medical records with incomplete data, patients transferred to other hospital services. Results: There was a higher prevalence of: mechanism of trauma due to motorcycle accidents (27.9%), surgical treatment (92.7%), with a higher frequency of use of plates and screws (68.5%). The most common type of fracture were bimalleolar and trimalleolar (23.8%; 20.5%), classified as AO44B2 and AO44B3, both with 17.8%. Conclusion: Ankle fractures in this study were predominantly seen in male patients, aged from 30 to 39 years, with the main mechanism of injury being a motorcycle accident. There was a correlation between mechanism of injury and fracture classification , with the most common being types 44B2 and 44B3, and the use of an external fixator in 9.1% of cases. The death rate was significant when related to associated injuries, in polyfractured patients. *Level of Evidence II, Retrospective study.*

## RESUMO

Objetivo: Avaliar o perfil epidemiológico dos pacientes com diagnóstico de fratura de tornozelo, internados em um hospital de nível terciário. Métodos: Estudo Observacional Transversal Retrospectivo. Critérios de Inclusão: Indivíduos com idade ≥18 (dezoito) anos, com diagnóstico de fratura de tornozelo, que realizaram tratamento cirúrgico e/ou conservador em um hospital terciário da cidade de São Paulo. Critérios de Exclusão: Prontuários com dados incompletos, pacientes transferidos para outros serviços hospitalares. Resultados: Encontrou-se maior prevalência de: mecanismo de trauma por acidente motociclístico (27,9%), tratamento cirúrgico (92,7%), com maior frequência de utilização de placas e parafusos (68,5%), tipo de fratura mais comum foram do bimaleolar e trimaleolar (23,8%; 20,5%), classificação por AO44B2 e AO44B3, ambas com 17,8%. Conclusão: As fraturas de tornozelo neste estudo mostraram-se de forma predominante em pacientes do sexo masculino, entre faixa etária de 30 e 39 anos, sendo o principal mecanismo de trauma o acidente motociclístico, Houve correlação entre mecanismo de trauma e classificação da fratura, sendo as mais presentes as do tipo 44B2 e 44B3, e utilização de fixador externo em 9,1% dos casos. O índice de óbito apresentou significância quando relacionado às lesões associadas, em pacientes polifraturados. *Nível de Evidência II, Estudo retrospectivo.*

## INTRODUCTION

 Ankle fractures correspond to 10% of all fractures in the human body, making the ankle the second most affected joint of the lower limbs, only behind hip fractures. ^1^ Recent studies conducted in the United States indicate that, between the years 1970 and 2000, there was an increase in tibiotarsal joint fractures among the bone injuries treated surgically, being diagnosed in 8.3 cases in 1000 medical consultations. ^2^

 Ankle fractures have a double peak of incidence, being frequent in young male adults, mostly due to sports injuries or traffic accidents, and in older female patients due to falls. ^3^ ^,^ ^4^

 The classification system of ankle fractures according to the Arbeitsgemeinschaft für Osteosynthesefragen (AO) group is based on the syndesmosis region level (A: infrasyndesmotic, B: transsyndesmotic, C: suprasyndesmotic) associated with subtypes according to the fracture degree. ^5^ ^,^ ^6^ For fractures of the articular surface of the distal third of the tibia, the Rüedi-Allgöwer system defines types I, II or III based on articular displacement and the degree of comminution. ^7^

 Despite the high incidence of this type of injury, there are still many conflicts in the literature on what treatment to perform. Choosing the appropriate treatment is essential to achieve fracture stabilization and consequently consolidation. Studies show that poorly stabilized fractures have a higher risk of new injury in addition to functional damage. ^5^ ^,^ ^8^

In view of the scarcity of national studies related to the epidemiological profile of patients with ankle fractures, the exponential growth of this type of injury, and its impact on quality of life, it is essential to conduct studies on the matter. Thus, this study objective is to trace the epidemiological profile of ankle fractures treated in a tertiary level hospital, to verify the relation between mechanism of injury and age, type of treatment, and prevalence of complications after ankle fractures.

## METHODS

This was a retrospective cross-sectional study. The Electronic Patient Records (EPR) system was used to search for patients with ankle fractures in the hospital admission records who were hospitalized and treated conservatively and surgically, in the period from January 2019 to July 2022. The following variables were analyzed: gender, age, mechanism of injury, type of fracture, fracture classification, associated injuries, previous pathologies, period of hospital stay, type of treatment, complications, and death. Medical records with incomplete data, patients aged under 18 years, or those transferred to other hospital services were excluded.

All the information used for this project was collected after the participants signed the Informed Consent Form (ICF), issued by the research team.

This study was registered and approved by the Research Ethics Committee registered in Plataforma Brasil, under CAEE number: 64374322.0.0000.5505

### Statistical Methods

Data were presented descriptively, with frequency and percentage for categorical variables, and with measure and standard deviation (SD) for continuous variables. Data normality was assessed using the Shapiro-Wilk test. The Chi-square test and Cramer’s V² test were used to verify the influence of age, gender, mechanism of injury, associated injuries, and type of fracture, previous pathologies, types of treatment, complications, period of hospital stay, and death, as well as the relation between these variables. A 0.05% significance level was used for all tests (p < 0.05). Statistical analyses were performed using the SPSS program (version 20.0).

## RESULTS

 A total of 245 medical records were selected, of which 35 were excluded for not meeting the inclusion criteria. Thus, the sample consisted of 219 individuals, 143 males (65.3%) and 76 females (34.7%), with a gender ratio of 1.8:1, individuals ranging from 18 to 75 years of age, with a mean age of 41.87 years and standard deviation (SD) of 14.41. Period of hospital stay with a minimum variation of one day and a maximum of 107 days, with a mean of 7.10 days, and standard deviation (SD) of 10.20 ( Table 1 ). 

**Table 1.** Sample characterization

VARIABLES	DESCRIPTION	FREQUENCY (f)	MALE	FEMALE	TOTAL (%)
GENDER		219	143 (65.3%)	76 (34.7%)	219 (100%)
AGE	18–75 YEARS OLD		µ 41.93 years old SD 14.41	µ 41.86 years old SD 14.37	µ 41.87 years old SD 14.41
PERIOD OF HOSPITAL STAY	Minimum: 1 day / Maximum: 107 days				µ 7.10 SD 10.20
AFFECTED SIDE	Right	91	62 (28.3%)	29 (13.2%)	41.5%
	Left	127	80 (36.5%)	47 (21.5%)	58%
	Bilateral	1	1 (0.5%)	0 (0%)	0.5%
MECHANISM OF INJURY	Car Accident	3	2 (0.9%)	1 (0.5%)	1.4%
	Motorcycle Accident	61	54 (24.7%)	7 (3.2%)	27.9%
	Physical Aggression	2	2 (0.9%)	0 (0%)	0.9%
	Run over	17	11 (5.0%)	6 (2.7)	7.8%
	Contusion	7	6 (2.7%)	1 (0.5%)	3.2%
	Sprain	60	27 (12.3%)	33 (15.1%)	27.4%
	Sprain during sport	6	5 (2.3%)	1 (0.5%)	2.7%
	Firearm injury	1	1 (0.5%)	0 (0%)	0.5%
	Fall from own height	27	13 (5.9%)	14 (6.4%)	12.3%
	Fall from height	25	14 (6.4%)	11 (5.0%)	11.4%
	Fall during sport	10	8 (3.7%)	2 (0.9%)	4.6%
TYPES OF FRACTURE	Lateral unimalleolar	53	35 (16%)	18 (8.2%)	24.2%
	Medial Unimalleolar	22	20 (9.1%)	2 (0.9%)	10.0%
	Posterior Unimalleolar	2	2 (0.9%)	0 (0%)	0.9%
	Bimalleolar (lateral + medial)	49	28 (12.8%)	21 (9.6%)	22.4%
	Bimalleolar (lateral + posterior)	3	2 (0.9%)	1 (0.5%)	1.4%
	Trimalleolar	45	22 (10.0%)	23 (10.5%)	20.5%
	Tibial Pilon	43	33 (15.1%)	10 (4.6%)	19.6%
	Tibial Pilon + Malleolar Fracture	2	1 (0.5%)	1 (0.5%)	0.9%
FRACTURE CLASSIFICATION	MALLEOLAR FRACTURE ACCORDING TO AO	174			79.5%
	AO 43B1	17	16 (7.3%)	1 (0.5%)	7.8%
	AO 44A1	2	1 (0.5%)	1 (0.5%)	0.9%
	AO 44A2	7	5 (2.3%)	2 (0.9%)	3.2%
	AO 44A3	2	1 (0.5%)	1 (0.5%)	0.9%
	AO 44B1	22	13 (5.9%)	9 (4.1%)	10%
	AO 44B2	39	18 (8.2%)	21 (9.6%)	17.8%
	AO 44B3	39	21 (9.6%)	18 (8.2%)	17.8%
	AO 44C1	22	15 (6.8%)	7 (3.2%)	10%
	AO 44C2	17	14 (6.4%)	3 (1.4%)	7.8%
	AO 44C3	7	5 (2.3%)	2 (0.9%)	3.2%
	TIBIAL PILON FRACTURE	43			19.6%
	Rüedi-Allgöwer Type I	5	3 (1.4%)	2 (0.9%)	3.2%
	Rüedi-Allgöwer Type II	16	11 (5.0%)	5 (2.3%)	7.3%
	Rüedi-Allgöwer Type III	21	18 (8.2%)	3 (1.4%)	9.6%
	Rüedi-Allgöwer Types I e II	1	1 (0.5%)	0 (0%)	0.5%
	PILON FRACTURE ASSOCIATED WITH MALLEOLAR FRACTURE	2			0.9%
	Rüedi-Allgöwer Type II / AO 43B1	1	1 (0.5%)	0 (0%)	0.5%
	Rüedi-Allgöwer Type II / AO 43B1	1	0 (0%)	1 (0.5%)	0.5%
ASSOCIATED INJURIES	Yes	117	84 (38.4%)	33 (15.1%)	53.4%
	No	102	59 (26.9%)	43 (19.6%)	46.6%
TYPE OF TREATMENT	Conservative	16	11 (5.0%)	5 (2.3%)	7.3%
	Surgical	203	132 (60.3%)	71 (32.4%)	92.7%
OPEN FRACTURE	Yes	33	26 (11.9%)	7 (3.2%)	15.1%
	No	186	117 (53.4%)	69 (31.5%)	84.9%
USE OF EXTERNAL FIXATOR	Yes	57	44 (20.1%)	13 (5.9%)	26.0%
	No	162	99 (45.2%)	63 (28.8%)	74.0%
COMPLICATIONS	IN-HOSPITAL	5			2.3%
	Hemodynamic alterations	2	1 (0.5%)	1 (0.5%)	0.9%
	Osteoarthritis	1	1 (0.5%)	0 (0%)	0.5%
	Inadequate soft tissues	1	1 (0.5%)	0 (0%)	0.5%
	Cellulite	1	1 (0.5%)	0 (0%)	0.5%
	POST-DISCHARGE	19			8.7%
	Surgical wound infection	8	8 (3.7%)	0 (0%)	3.7%
	Injury of the syndesmosis	2	2 (0.9%)	0 (0%)	0.9%
	Osteomyelitis	2	2 (0.9%)	0 (0%)	0.9%
	Nonunion	1	1 (0.5%)	0 (0%)	0.5%
	Pseudoarthrosis	1	1 (0.5%)	0 (0%)	0.5%
	Other	5	4 (1.8%)	1 (0.5%)	2.3%
PREVIOUS DISEASES					56.6%
	Diabetes Mellitus	17	10 (10.5%)	7 (7.4%)	17.9%
	Drug addiction	15	15 (15.8%)	0 (0%)	15.8%
	Alcoholism	12	12 (12.6%)	0 (0%)	12.6%
	Systemic arterial hypertension	25	14 (14.7%)	11 (11.6%)	26.3%
	Obesity	6	3 (3.2%)	3 (3.2%)	6.4%
	Smoking habit	22	20 (21.1%)	2 (2.1%)	23.2%
	Other	70	41 (43.2%)	29 (30.5%)	73.7%
READMISSION	Yes	21	19 (8.7%)	2 (0.9%)	9.6%
	No	198	124 (56.6%)	74 (33.8%)	90.4%
DISCHARGE CONDITIONS	Improved	211	136 (62.1%)	75 (34.2%)	96.3%
	Other	8	7 (3.2%)	1 (0.5%)	3.7%
DEATH	Yes	3	3 (1.4%)	0 (0.0%)	1.4%
	No	216	140 (63.9%)	76 (34.7%)	98.6%

Source: Electronic Patient Record System (EHR) – HSP

This study result shows a higher prevalence of fractures in males, aged from 30 to 39 years, corresponding to 18.7% of the cases. Females had a higher prevalence from 40–49 years and 60 years of age or older, both represented by 7.8%.

 As illustrated in Table 2.1 , the most common associated mechanism in young patients was motorcycle accidents, as 27.9% of the cases among patients from 30 to 39 years of age (p < 0.001; V² Cramer 0.32). 

 The most evident types of fracture were bimalleolar and trimalleolar, totaling 44.3% of the sample. Regarding the analysis of the fractures classification by OA, the most common was type 44B as 45.6% of the total sample. The most predominant subtypes were B2 and B3, both with a frequency of 39 (17.8%) individuals. Table 2.2 shows a higher incidence of this classification when related to the mechanism of injury, with sprains causing 19 (8.7%) cases (p = 0.014; V ^2^ Cramer 0.26). For tibial pilon fractures, which were 19.6% of the sample, the Rüedi-Allgöwer classification was used to determine type III fractures (9.6%), type II (7.3%), and type I (2.3%). 

 Regarding the treatment performed, 203 (92.7%) of the patients underwent surgical treatment, while 16 (7.3%) patients were conservatively treated. The most commonly applied form of surgical intervention was stabilization of fractures with plate and screw (68.5%; Table 3.1 ). The use of external fixators comprises 26% of the sample, and 9.6% in the definitive treatment. According to the results in Table 3.2 , its use can be observed in 9.1% of motorcycle accidents and 3.2% of injuries caused by sprains (p0.003; V ^2^ Cramer 0.36). 

 A total of 176 (79.7%) individuals with associated injuries were verified, with the highest rate for ankle dislocations (41.10%). The result related to death can be observed in patients who also had associated injuries, represented by p < 0.001 e V ^2^ Cramer 0.81, with the highest frequency for polytraumas ( Table 4 ). 

**Table 2.1.** Mechanism of injury versus Age.

	18 TO 29 YEARS OLD	30 TO 39 YEARS OLD	40 TO 49 YEARS OLD	50 TO 59 YEARS OLD	60 YEARS OR OLDER	P-VALUE	V ^2^ CRAMER (P-VALUE)	TOTAL
						<0.001	0.325	
MECHANISM OF INJURY								
Car Accident	2	0	0	0	1			3 (1.4%)
Motorcycle Accident	22	24	12	2	1			61 (27.9%)
Physical Aggression	0	0	1	0	1			2 (0.9%)
Run over	2	4	2	5	4			17 (7.8%)
Contusion	1	2	1	2	1			7 (3.20%)
Sprain	6	17	13	13	11			60 (27.4%)
Sprains during Sport	4	1	1	0	0			6 (2.7%)
Firearm injury	0	0	0	1	0			1 (0.5%)
Fall from own height	4	3	5	6	9			27 (12.4%)
Fall from height	3	2	9	6	5			25 (11.4%)
Fall during Sport	6	0	3	1	0			10 (4.6%)
TOTAL (n:%)	50 (22.8%)	53 (24.2%)	47 (21.5%)	36 (16.4%)	33 (15.1%)			219 (100%)

Source: Electronic Patient Records (EPR) system – HSP

**Table 2.2.** Mechanism of Injury versus Classification by AO.

CLASSIFICATION BY AO	44B1	44A1	44A2	44A3	44B1	44B2	44B3	44C1	44C2	44C3	OTHER	P-VALUE	V ^2^ CRAMER (P-VALUE)	TOTAL (f %)
												0.014	0.266	
MECHANISM OF INJURY														
Car Accident	0	0	1	0	0	1	0	0	0	0	1			3 (1.4%)
Motorcycle Accident	11	1	3	0	2	6	6	7	7	1	17			61 (27.9%)
Physical Aggression	0	0	0	0	2	0	0	0	0	0	0			2 (0.9%)
Run over	2	0	0	0	1	2	2	1	4	1	4			17 (7.8%)
Contusion	1	0	0	0	0	2	2	2	0	0	0			7 (3.2%)
Sprain	2	1	0	2	9	16	19	5	3	2	1			60 (27.4%)
Sprains during Sport	0	0	0	0	0	2	1	2	1	0	0			6 (2.7%)
Firearm injury	0	0	0	0	0	0	0	0	0	0	1			1 (0.5%)
Fall from own height	1	0	1	0	5	6	7	2	0	1	4			27 (12.3%)
Fall from height	1	0	1	0	1	3	3	1	0	0	15			25 (11.4%)
Fall during Sport	0	0	1	0	2	0	1	2	2	2	0			10 (4.6%)
TOTAL (f %)	18	2	7	2	22	38	41	22	17	7	43			219 (100%)

Source: Electronic Patient Records (EPR) system – HSP

**Table 3.1.** Types of Definitive Treatment.

		MALE	(%)	FEMALE	(%)	TOTAL (f %)
CONSERVATIVE		11	5	5	2.3	16 (7.3%)
SURGICAL	Tension band	3	1.4	0	0	3 (1.4%)
	External Trans. Delta Fix.	8	3.7	3	1.4	11 (5.0%)
	External Ilizarov Fix.	9	4.1	1	0.5	10 (4.6%)
	HIMB	4	1.8	0	0	4 (1.8%)
	Screws	20	9.1	2	0.9	22 (10%)
	Plate and Screws	85	38.8	65	29.7	150 (68.5%)
	Bridge plate	3	1.4	0	0	3 (1.4%)
TOTAL (n:%)		143	65.3	76	34.8	219 (100%)

Source: Electronic Patient Records (EPR) system – HSP

Abbreviations:

Fix. Fixator Trans Transarticular HIMB Intramedullary Interlocking Nail

**Table 3.2.** Mechanism of injury versus External Fixator.

	NO	YES	P-VALUE	V ^2^ CRAMER (P-VALUE)	TOTAL
			0.003	0.367	
MECHANISM OF INJURY					
Car Accident	1 (0.5%)	2 (0.9%)			3 (1.4%)
Motorcycle Accident	41 (18.7%)	20 (9.1%)			61 (27.9%)
Physical Aggression	2 (0.9%)	0 (0.0%)			2 (0.9%)
Run over	9 (4.1%)	8 (3.7%)			17 (7.8%)
Contusion	6 (2.7%)	1 (0.5%)			7 (3.2%)
Sprain	53 (24.2%)	7 (3.2%)			60 (27.4%)
Sprains during Sport	5 (2.3%)	1 (0.5%)			6 (2.7%)
Firearm injury	0 (0.0%)	1 (0.5%)			1 (0.5%)
Fall from own height	21 (9.6%)	6 (2.7%)			27 (12.3%)
Fall from height	15 (6.8%)	10 (4.6%)			25 (11.4%)
Fall during Sport	9 (4.1%)	1 (0.5%)			10 (4.6%)
TOTAL (n:%)	162 (74%)	57 (26%)			219 (100%)

Source: Electronic Patient Records (EPR) system – HSP

**Table 4.** Death versus associated injuries.

	NO	YES	P-VALUE	V ^2^ CRAMER (P-VALUE)	TOTAL
			<0.001	0.816	
MECHANISM OF INJURY					
Abrasions	1	0			1 (0.5%)
Fracture of the upper limbs	5	0			5 (2.3%)
Fracture of the lower limbs	37	0			37 (16.9%)
Fracture of the upper limbs/lower limbs	5	0			5 (2.3%)
Fractures of the lower limbs and others	4	0			4 (1.8%)
Other Fractures	1	0			1 (0.5%)
Ligament Injury	6	0			6 (2.7%)
Injury of the syndesmosis	3	0			3 (1.4%)
Ligament Injury + Injury of the syndesmosis	1	0			1 (0.5%)
Ankle Dislocation	89	1			90 (41.1%)
Ankle Dislocation + Other Fractures	4	0			4 (1.8%)
Ankle Dislocation + Dislocation of the syndesmosis	1	0			1 (0.5%)
Ankle Dislocation + Ligament Injury	1	0			1 (0.5%)
Ankle Subluxation	2	0			2 (0.9%)
Other Dislocations	2	0			2 (0.9%)
Polyfractures	8	2			10 (4.7%)
No associated injuries	46	0			46 (21.0%)
TOTAL (n:%)	216	3			219 (100%)

Source: Electronic Patient Records (EPR) system – HSP

Abbreviations:

MMSS Upper Limbs MMII Lower Limbs

Previous pathologies were present in 43.4% of the total sample. The diseases with the highest incidences were systemic arterial hypertension (SAH) (26.3%), followed by smoking habits and Diabetes Mellitus (DM) (23.2% and 17.9%, respectively). Complications during the hospital stay occurred in 2.3% of the sample, with prevalence of hemodynamic alterations (40%). In the post-discharge period, complications could be observed in 8.7% of the sample, of which 42.1% were surgical wound infection.

## DISCUSSION

 Ankle fractures have been growing annually and can cause disability, decreased productivity, time off work, and compromised quality of life, generating significant costs for health systems ^8^ ^,^ ^9^ . 

A total of 76 women and 143 men participated in this study, from 18 to 75 years of age, with a mean age of 41.87 years.

The most observed mechanism of injury was motorcycle accidents (27.9%) followed by ankle sprains (27.4%).

 There was a correlation between the mechanism of injury and age, with motorcycle accidents being more frequent in young individuals, aged from 30 to 39 years (p < 0.001). This index can be attributed to multifactorial conditions, such as greater use of this vehicle as a work tool, driver vulnerability, and traffic conditions. ^9^ ^,^ ^10^ In the studies by Silva et al. ^10^ and by Santos et al., ^11^ the representation of young people injured in motorcycle accidents was 83.5% and 87.43%, respectively. 

 Traffic accident prevention policies, such as education and investing in infrastructure, should be implemented to seek the reduction of accidents incidence. Despite the technological evolution of automobiles, with life protection measures, such as the installation of airbags, among other technologies, the incidence of fractures remains high, as well as these injuries severity, which require greater care and treatment time. ^12^

 This study shows that 53.4% of these patients had other associated injuries, with ankle dislocation being the most observed (41.1%), followed by lower limb trauma. These data corroborate the findings of Debieux et al, ^13^ which pointed out that, of the 387 patients who suffered motorcycle accidents in a 16-month period, 16% of them had foot fractures and 12.7% ankle fractures. Associated lower limb injuries contribute to longer hospital stays and higher morbidity rates. ^10^ ^,^ ^11^ In our findings, there was a statistical correlation between multiple trauma patients and death (p < 0,001), which may be related to the severity and the mechanism of the injury, since high-energy trauma injuries are associated with severe sequelae. ^12^

 A recent study conducted in a hospital in the city of São Paulo found a high incidence of ankle fractures in older women, possibly due to postmenopausal osteoporosis. ^14^ This study showed a higher number of fractures in women aged 60 years or older, but the collected data were not statistically significant. The study carried out by Newman ^15^ et al. included patients who were admitted to emergency services in the United States and obtained a sample with a mean age of 37 years, with predominance of females (56%). The most observed mechanism of injury was falls from height (54%), followed by sports injuries (20.76%). The most affected age group was the one from 10 to 19 years, while women were more affected in the other age groups. 

 This study had a high rate of open fractures (15.1%), which corroborate the data presented by Mizusaki et al. ^16^ The author also states that the high number of open fractures is related to the large number of motorcycle accidents occurring near the hospital where the study was conducted. 

 The use of fixators in our study was significant when related to the mechanism of injury (p = 0.003) and was observed in 9.1% of motorcycle accidents. It is worth remembering that this form of accident is considered high energy trauma and of great exposure. ^9^ ^,^ ^10^ ^,^ ^12^

 This study showed a large number of surgically treated patients, totaling 203 (92.7%). For Mizusaki et al., ^16^ the submission of patients to surgical treatment is not necessarily consistent with the success of the results, since these patients are exposed to additional complications. ^17^ In that study, only 25.34% of the individuals were treated conservatively, which demonstrates that surgical intervention is the most preferred. 

 The evolution of synthesis materials and operative techniques increases the treatment success rate. Surgical treatment is usually indicated for cases of the following types of fractures: AO44A1; AO44A2; AO44A3; AO44B1 and all above AO44B1.2. ^18^ Surgical stabilization is indicated for injuries with risk of fracture fragments displacement and joint involvement. 

 There was a correlation between the classification of the fracture and the mechanism of injury (p = 0.014), since the classifications AO44B2 and AO44B3 corresponded to 35.6% of the sample and, according to studies, subtype B is the most described in the national literature, considered a type of serious injury. ^18^ ^,^ ^19^

 In this study, there was a predominance of bimalleolar (23.8%) and trimalleolar (20.5%) fractures, differing from the data of Misuzaki et al., ^16^ who reported in their research 83.33% unimalleolar fractures, 44.67% in the lateral malleolus, 38.66% in the medial malleolus, and 10% and 6.67% of bimalleolar and trimalleolar fractures, respectively. As the degree of the fracture increases, there is a worsening of the prognosis. ^16^ ^,^ ^19^ ^-^ ^21^ Tibial pylon fractures were observed in 20.6% (45 cases), of which most were type III fractures (21 cases; 46.7%). This classification is considered severe because it involves major soft tissue involvement. ^19^ Zelle et al. ^21^ mentions that patients affected by high-energy fractures tend to present functional impairments in addition to developing chronic diseases. 

 In this study, complications during hospitalization occurred in 5 patients (2.28%) and in the post-discharge period it occurred in 19 patients (8.17%). The most prevalent were those characterized as acute, such as surgical wound infection (42.10%) and osteomyelitis (10.5%), which were more frequently observed in more complex cases. For Hu et al., ^22^ the emergence of postoperative infections may be attributed to factors such as surgery time, type of incision, comorbidities, and type of fracture. The rate of complications in this study differs from the findings of Sakaki et a1., ^9^ who reported a high rate of infection (23.1%) among the studied patients. 

 Previous pathologies in this study were more observed in male patients (46.4% of the sample), with smoking habits and drug addiction being the most prevalent with 21.10% and 15.8%, respectively. These findings endorse the studies by Santos et al., ^23^ which addresses the of these individuals exposure to major risk factors and behavioral influence. Among women, it was observed that systemic arterial hypertension (SAH) and diabetes mellitus (DM) were present in a higher percentage (11.6%; 7.4%). It is important to emphasize that diabetic patients have an increased risk of postoperative infection due to the impairment of the distal segments perfusion, impairing blood supply and, consequently, healing. ^22^

There are few epidemiological studies of ankle fractures published in the national literature, and this type of study is extremely important to verify the main affected population and the mechanism of injury, in order to develop prevention policies, as well as to map the most performed type of treatment and period of hospitalization, important data for the health system, and hospital management.

In addition to the scarcity of information from national studies regarding the epidemiological survey of ankle fractures, this research had a information collection limitation due to the lack of a protocol for resident physicians to fill in data.

## CONCLUSION

The sample consisted of 219 individuals, 143 males (65.3%) and 76 females (34.7%), with a mean age of 41.87 years, and a mean period of hospital stay of 7.10 days.

In this study, ankle fractures were predominant in male patients from 30 to 39 years of age, with the main mechanism of injury being motorcycle accidents. There was a correlation between the mechanism of injury and fracture classification, with the most common types being 44B2 and 44B3, and the use of external fixators in 9.1% of the cases. The death rate was significant when related to associated injuries in polyfractured patients.

Most of the treatment intervention was surgical (92.7%), with the use of plates and screws (29.7%).

## References

[1] Seewoonarain S., Prempeh M., Shakokani M., Magan A. (2016). Ankle Fractures: Review Article. J Arthritis.

[2] Scheer R. C., Newman J. M., Zhou J. J., Oommen A. J., Naziri Q., Shah N. V. (2020). Ankle Fracture Epidemiology in the United States: Patient-Related Trends and Mechanisms of Injury. J Foot Ankle Res.

[3] Piccini C. F., Endres G., Prado J. M., Scherer M. B. (2018). Epidemiological profile of patients undergoing surgical treatment of ankle fractures in a tertiary hospital. Sci J Foot Ankle.

[4] Court-Brown C. M., Caesar B. (2006). Epidemiology of adult fractures: A review. Injury.

[5] Barei D. P., Court-Brown C. M., Heckman J. D., Mcqueen M. M., Ricci W. M., Tornetta P. (2017). Fraturas em adultos: de Rockwood e Green.

[6] Briet J. P., Hietbrink F., Smeeing D. P., Dijkgraaf M. G. W., Verleisdonk E. J., Houwert R. M. (2019). Ankle Fracture Classification: An Innovative System for Describing Ankle Fractures. J Foot Surg.

[7] Ramos L. C., Gonçalves H. M., Freitas A., Oliveira M. P., Lima D. M. S., Carmargo W. S. (2021). Evaluation of the Reproducibility of Lauge-Hansen, Danis-Weber and AO Classifications for Ankle Fractures. Rev Bras Ortop.

[8] Wu A. M., Bisignano C., James S. L., Abady G. G., Abedi A., Abu-Gharbieh E. (2021). Global, regional, and national burden of bone fractures in 204 countries and territories, 1990-2019: a systematic analysis from the Global Burden of Disease Study 2019. Lancet Healthy Longev.

[9] Batista F. S., Silveira L. O., Castillo J. J. A., Pontes J. E., Villalobos L. D. C. (2015). Epidemiological profile of extremity fractures in victims of motorcycle accidents. Acta ortop. bras.

[10] Silva D. W., Andrade S. M., Soares D. A., Soares D. F. P. P., Mathias T. A. F. (2008). Perfil do trabalho e acidentes de trânsito entre motociclistas de entregas em dois municípios de médio porte do estado do Paraná, Brasil. Cad. Saúde Pública.

[11] Santos A. M. R., Moura M. E. B., Nunes B. M. V. T., Leal C. F. S., Teles J. B. M. (2008). Profile of motorcycle accident victims treated at a public hospital emergency department Cad. Saúde Pública.

[12] Richter M., Otte D., Jahanyar K., Blauth M. (2000). Upper extremity fractures in restrained front-seat occupants. J Trauma.

[13] Debieux P., Chertman C., Mansur N. S. B., Dobashi E., Fernandes H. J. A. (2010). Lesões do aparelho locomotor nos acidentes com motocicleta. Acta ortop. bras.

[14] Stéfani K. C., Pereira M. V., Lago R. R. (2017). Epidemiological study of foot and ankle fractures among Civil Servants in the State of São Paulo. Rev ABTPé.

[15] Scherr R. C., Newman J. M., Zhou J. J., Oommen A. J., Naziri Q., Shah N. V. (2020). Ankle Fracture Epidemiology in the United States: Patient-Related Trends and Mechanisms of Injury. J Foot Ankle Surg.

[16] Mizusaki J. M., Prata S. D. S., Rizzo M. A. G., Gonzaga L. A. S., Carneiro L. P. (2021). Epidemiological study of ankle fractures. J Foot Ankle.

[17] Chen X., Lim J. A., Zhou A., Thahir A. (2021). Currents concepts of the perioperative management of closed ankle fractures. J Perioper Pract.

[18] Goost H., Wimmer M. D., Barg A., Kabir K., Valdebarrano V., Burger C. (2014). Fractures of the ankle joint: investigation and treatment options. Dtsch Arztebl Int.

[19] Sakaki M. H., Matsumura B. A. R., Dotta T. A. G., Pontin P. A., Santos A. L. G., Fernades T. D. (2014). Epidemiologic study of ankle fractures in a tertiary hospital. Acta ortop. bras.

[20] Juto H., Nilsson H., Morberg P. (2018). Epidemiology of Adult Ankle Fractures: 1756 cases identified in Norrbotten County during 2009-2013 and classified according to AO/OTA. BMC Musculoskelet Disord.

[21] Zelle B. A., Dang K. H., Ornell S. S. (2019). High-energy tibial pilon fractures: an instructional review. Int Orthop.

[22] Hu H., Zhang J., Xie X. G., Dai Y. K., Huang X. (2022). Identification of risk factors for surgical site infection after type II and type III tibial pilon fracture surgery. World J Clin Cases.

[23] Santos L. F. S., Fonseca J. M. A., Cavalcante B. L. S., Lima C. M. (2016). Estudo epidemiológico do trauma ortopédico em um serviço público de emergência. Cad. Saúde Colet.

